# Geographic coincidence of increased malaria transmission hazard and vulnerability occurring at the periphery of two Tanzanian villages

**DOI:** 10.1186/1475-2875-12-24

**Published:** 2013-01-18

**Authors:** Tanya L Russell, Dickson W Lwetoijera, Bart GJ Knols, Willem Takken, Gerry F Killeen, Louise A Kelly-Hope

**Affiliations:** 1 Ifakara Health Institute, Environmental Sciences Thematic Group, Ifakara, Tanzania; 2 Liverpool School of Tropical Medicine, Vector Group, Pembroke Place, Liverpool, UK; 3 Faculty of Medicine, Health and Molecular Sciences, James Cook University, Cairns, Australia; 4 In2Care BV, Costerweg 5, Wageningen, 6702 AA, The Netherlands; 5 Laboratory of Entomology, Wageningen University and Research Centre, Wageningen, The Netherlands; 6 Liverpool School of Tropical Medicine, Centre for Neglected Tropical Diseases, Pembroke Place, Liverpool, UK

## Abstract

**Background:**

The goal of malaria elimination necessitates an improved understanding of any fine-scale geographic variations in transmission risk so that complementary vector control tools can be integrated into current vector control programmes as supplementary measures that are spatially targeted to maximize impact upon residual transmission. This study examines the distribution of host-seeking malaria vectors at households within two villages in rural Tanzania.

**Methods:**

Host-seeking mosquitoes were sampled from 72 randomly selected households in two villages on a monthly basis throughout 2008 using CDC light-traps placed beside occupied nets. Spatial autocorrelation in the dataset was examined using the Moran’s *I* statistic and the location of any clusters was identified using the Getis-Ord Gi* statistic. Statistical associations between the household characteristics and clusters of mosquitoes were assessed using a generalized linear model for each species.

**Results:**

For both *Anopheles gambiae* sensu lato and *Anopheles funestus,* the density of host-seeking females was spatially autocorrelated, or clustered. For both species, houses with low densities were clustered in the semi-urban village centre while houses with high densities were clustered in the periphery of the villages. Clusters of houses with low or high densities of *An. gambiae* s.l. were influenced by the number of residents in nearby houses. The occurrence of high-density clusters of *An. gambiae* s.l. was associated with lower elevations while *An. funestus* was also associated with higher elevations. Distance from the village centre was also positively correlated with the number of household occupants and having houses constructed with open eaves.

**Conclusion:**

The results of the current study highlight that complementary vector control tools could be most effectively targeted to the periphery of villages where the households potentially have a higher hazard (mosquito densities) and vulnerability (open eaves and larger households) to malaria infection.

## Background

The frontline vector tools deployed in the battle against malaria transmission are long-lasting insecticidal nets (LLINs) and indoor residual spraying (IRS) [1,2]. These tools are highly effective and their use has led to a significant reduction of transmission in many parts of Africa, including places that were historically holoendemic [3-6]. In response to such success, the international community has now prioritized regional and national malaria elimination, with a long-term goal of malaria eradication [7]. However, vector control that solely targets insecticides to the inside of houses is unlikely to be sufficient to achieve elimination [8]. Thus there is a need for complementary vector control tools to target a range of alternative stages in the mosquito cycle, such as the larval stage, mating or sugar feeding. Such complementary tools will target specific ecosystems and understanding the fine-scale geographic variations in *Anopheles* mosquitoes and transmission risk will enable tools to be developed and effectively integrated into current vector control programmes. This need for detailed geographic research has been highlighted by recent literature demonstrating that malaria transmission is highly heterogeneous across the landscape [9-12].

Changes in malaria transmission risk can be measured by the entomological inoculation rate (EIR) [13,14], which is the product of the anopheline biting rate and the proportion of infectious females (sporozoite rate). With regard to the anopheline biting rate, household-level characteristics have been demonstrated to influence biting rates, such as the number of occupants, screened windows, closed eaves or ceilings [15-17]. Further, some level of geographic clustering has been observed where houses closest to breeding sites tend to experience higher adult biting rates [10,12,18-21] which is supported with mathematical modelling [9]. However, in peri-urban or rural situations where houses are spread over large distances (many kms) and often inter-dispersed with larval habitats, the heterogeneity of anopheline biting rates may be influenced by household characteristics in addition to distance from the nearest breeding site. Understanding the spatial clustering of anopheline biting rates at the household level is important because households are the focal point of many predictive malaria models and, most importantly, are perhaps the easiest of targets for delivery of vector control measures through either vertical or horizontal delivery strategies.

The current study therefore examines the geographic relationships of anopheline host-seeking patterns at a household and village level in East Africa. The null hypothesis tested was that the adult biting rate would be randomly distributed across the households within villages. For this analysis, household-level characteristics (elevation, the number of occupants, closed eaves, the presence of bed nets, and distance from the village centre) were taken into account.

## Methods

### Study area

**Figure 1:**
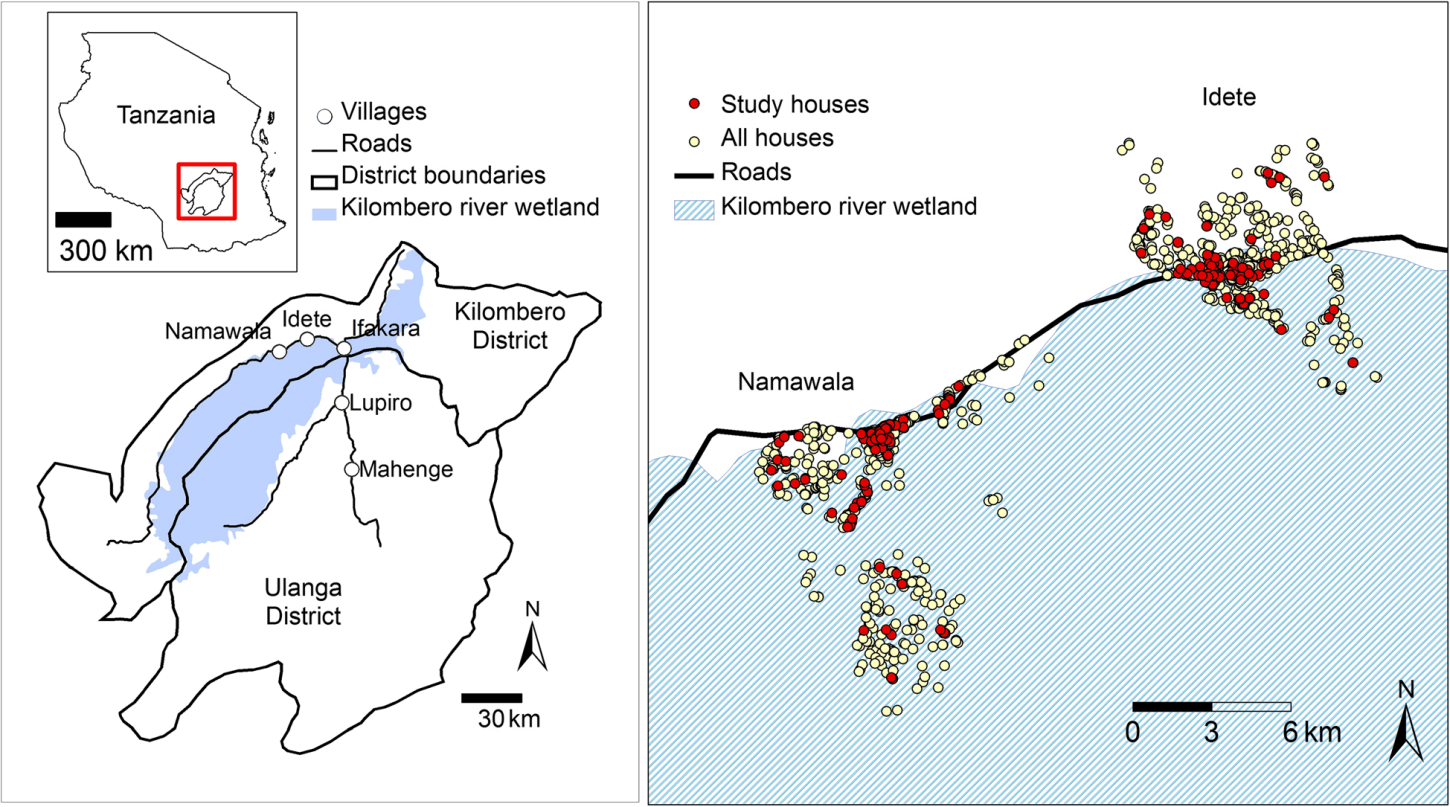
Kilombero and Ulanga districts (8.1°S and 36.6°E) in Tanzania showing Namawala and Idete villages (left) and spatial distribution of sentinel houses used for sampling the local anopheline population (right).

The study was conducted in the neighbouring villages of Namawala and Idete, located in the Kilombero Valley (8.1°S and 36.6°E), south-eastern Tanzania (Figure 1). These communities experience hyperendemic malaria transmission; primarily vectored by large populations of *Anopheles gambiae* sensu lato. In this area, this species complex has been historically represented by the two morphologically identical sibling species: *Anopheles gambiae* sensu stricto and *Anopheles arabiensis*[10,24] but it should be noted that the proportional contribution of the former has been dramatically reduced following scale up of LLINs in the area [24,25]. A third, locally important vector species is *Anopheles funestus* sensu stricto. The ecosystem is dominated by a low-lying river valley, 150 km long and up to 40 km wide, which is interdispersed with villages and rice farms. Annual rains (December to May) create large quantities of ephemeral aquatic habitat suitable for *An. gambiae* s.l. oviposition and larval development. Both villages have semi-urban town centres with many people also residing in the rural farming regions on the outskirts (Idete = 1,229 and Namawala = 767 households). Idete village has been constructed in a slightly more elevated area compared with Namawala (Figure 1). There are subtle cultural differences between the villages, with more pastoral farmers residing in Namawala; for example, during 2008, there were 306 head of cattle in Idete and 6,667 in Namawala [24].

### Mosquito population sampling

Seventy-two households were randomly selected for mosquito sampling in both villages using census information from the Ifakara Health Institute (IHI) Demographic Surveillance System (Figure 1). For each household, the location and elevation was recorded using a handheld GPS unit (eTrex, Vista, Garmin Inc, USA). The distance of each household from the village centre was calculated using ArcGIS 10 software (ESRI, Redlands, CA, USA). Additionally, the physical structure of the eave openings was recorded directly by observation. The use of bed nets (either LLINs or insecticide-treated nets [ITNs]) and the number of household occupants was recorded during a one-time survey of all household heads.

Each house was visited once a month (six houses/day, four days/week and three weeks/month) over a period of 12 months (January to December 2008). Mosquitoes were collected inside each house using one CDC light trap for 12 hours (7 pm to 7 am) [26]. The light trap, fitted with an incandescent bulb, was placed 1–1.5 m above the floor and close to the feet of an LLIN occupant [27]. The LLIN used was provided to each participating household by the study team (Olyset, A to Z Textile Mills Ltd, Tanzania). Although permethrin-treated bed nets exhibit modest excito-repellency, they have surprising little effect on the relative efficiency of light traps when compared with untreated bed nets [28,29]. Traps were inspected each morning and all mosquitoes were morphologically identified to species complex or group and classified by sex [30]. The sibling species identity of the *An. gambiae* s.l. complex was identified using PCR [31]. For both *An. gambiae* s.l. and *An. funestus*, any specimens that contained sporozoites in the salivary glands were identified using ELISA [32]. Owing to the large number of female mosquitoes caught per trapping effort (up to approx 1,500), separate random subsamples, each averaging approximately 10% of the total in each trap, were used for molecular analysis. In cases where the catch was less than 10 females, molecular analysis was conducted for all individuals. Prior to molecular analysis, mosquitoes were stored at −20°C in micro centrifuge tubes containing a small amount of silica drying agent. The age structure of the *An. gambiae* s.l. population was estimated using parity dissections on a subset of the samples caught [33].

### Spatial and statistical analysis

The spatial and statistical analyses were conducted for *An. gambiae* s.l. and *An. funestus*. Regarding *An. gambiae* s.l., the ratio of *An. arabiensis* to *An. gambiae* s.s. was examined with a binomial GLMM with a categorical explanatory variable for week and adjusted for multiple comparisons with Dunnett’s test.

The spatial patterns in the dataset were analysed using the geographical information systems software ArcGIS 10 with Spatial Analysis and Statistical Tools (ESRI, Redlands, CA, USA). To assess any spatial patterns over time, the 12-month sampling period was broken into four time periods: January to February, March to April, May to June and July to December [24]. The time periods were selected to reflect the temporality of the system where the mosquito densities undergo large fluctuations during the wet season (January to June; thus three divisions) with less variation during the dry season (July to December; thus one division). For each month, one light trap sample was collected from each household; to enable spatial analysis with each household being a point of interest, the total number of mosquitoes collected from all trapping efforts (within the specified time frame) were summed.

Initially, the spatial patterns of *An. gambiae* s.l. and *An. funestus* densities within the two villages were mapped. Next any spatial autocorrelation patterns, i e, clustered, dispersed, random, were analysed using the Moran’s *I* statistic [34]. Sequentially, the localities of clustered households with high or low anopheline densities were identified using the Getis-Ord Gi* statistic [35]. Statistically significant (at a level of 0.05) clusters of households with high densities of anophelines were identified with *Z* scores >1.96, or *vice versa*, clustered households with low densities of anophelines were identified with *Z* scores < −1.96. For all spatial analyses, the spatial relationship among houses was conceptualized using the inverse distance, which is most appropriate for continuous point datasets because closer houses have larger influences on the computations for each target house than houses that are further away. Also, the distance between neighbouring features was calculated using the Euclidean distance and were run separately for each village.

Next, any association between the location of anopheline clustering and household characteristics was investigated. In Namawala and Idete, there was a clear socio-economic gradient from the semi-urban centres towards the rural village outskirts, and thus there were potential correlations between the household characteristics with distance from the village centre. The correlation of household characteristics, which were ordinal data (the number of occupants and the number of bed nets per person), was investigated using Pearson’s correlation coefficient. The presence of eaves was a binary factor (open or closed), and the correlation of this parameter with distance was investigated with a generalized linear model (GLM) with a binomial distribution and a logit link function. Sequentially, statistical associations between the household characteristics and clusters of mosquitoes were assessed using a GLM for each species. To account for spatial autocorrelations, the GLM was run with the Getis-Ord Gi* *Z* Score as the dependent factor and with a normal distribution. The independent factors incorporated in the model were: elevation (m), the presence of eaves, the number of occupants, the number of bed nets per person and distance from the village centre (m). Interaction terms were included for any correlated independent variables. This analysis was conducted using R statistical software (ver.2.14.2).

### Ethics

Ethical approval for the study was obtained from the IHI Institutional Review Board (IHRDC/IRB/No. A-32) and the Medical Research Coordination Committee of the National Institute for Medical Research (NIMR/HQ/R.8a/Vol. IX/764) in Tanzania. When the study commenced, permission was obtained from each household owner who, after consenting, signed an informed consent form stating their willingness to participate in the study.

## Results

**Figure 2:**
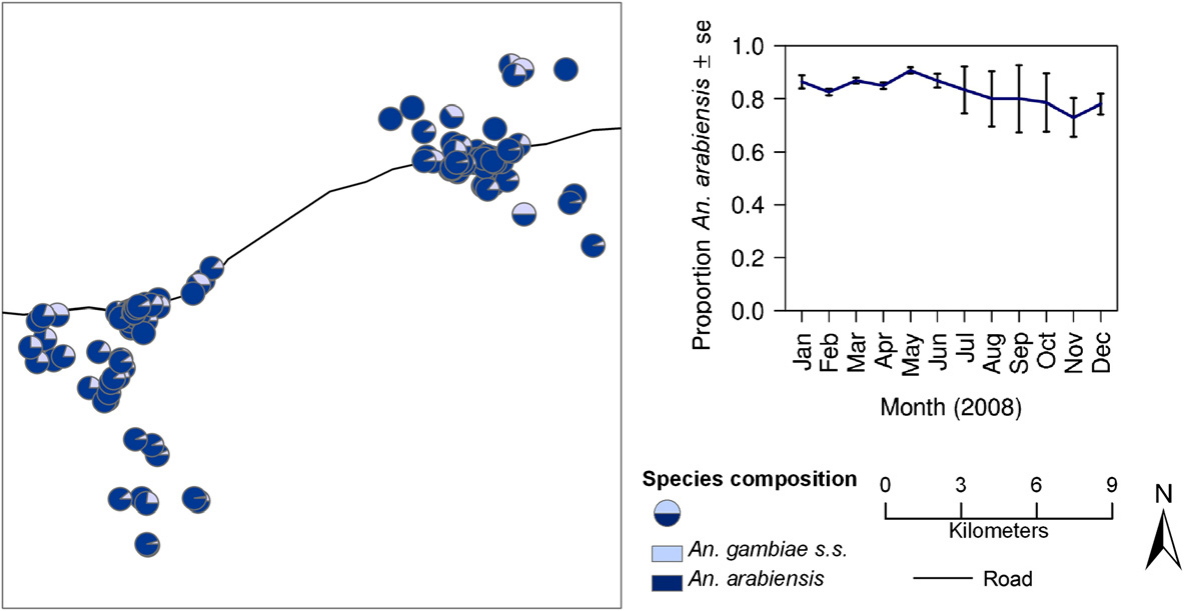
**Spatial and temporal distribution of the sibling species *Anopheles gambiae *s.s. and *Anopheles arabiensis*.**

During the 12-month, mosquito-sampling period, 1,648 light-trap nights of sampling were conducted. A total of 97,437 female mosquitoes were caught. Of these mosquitoes, 30.9% (*n* = 30,111) were *An. gambiae* s.l.*,* comprising 85.8% *Anopheles arabiensis* and 14.2% *An. gambiae* s.s. (*n* = 2,924 PCR amplifications). The remaining mosquitoes were 2.0% *An. funestus* (*n* = 1,950), 62.0% *Culex* spp (*n* = 60,442), 2.4% *Mansonia* spp (*n* = 2,302) and 2.7% other species including *Aedes* and *Coquillettidia* spp (*n* = 2,605). The ratio of *An. arabiensis* to *An. gambiae* s.s. remained constant throughout the study (binomial GLMM, *p* >0.05, Figure 2). Therefore the detailed spatial analysis was conducted for the species complex overall. The average number of *An. gambiae* s.l. per light-trap night was 18.3 ± 2.3 and for *An. funestus* was 1.2 ± 0.1. The parity rate was only calculated for *An. gambiae* s.l. and the portion of the population that were parous was 43% (397/916).

**Figure 3:**
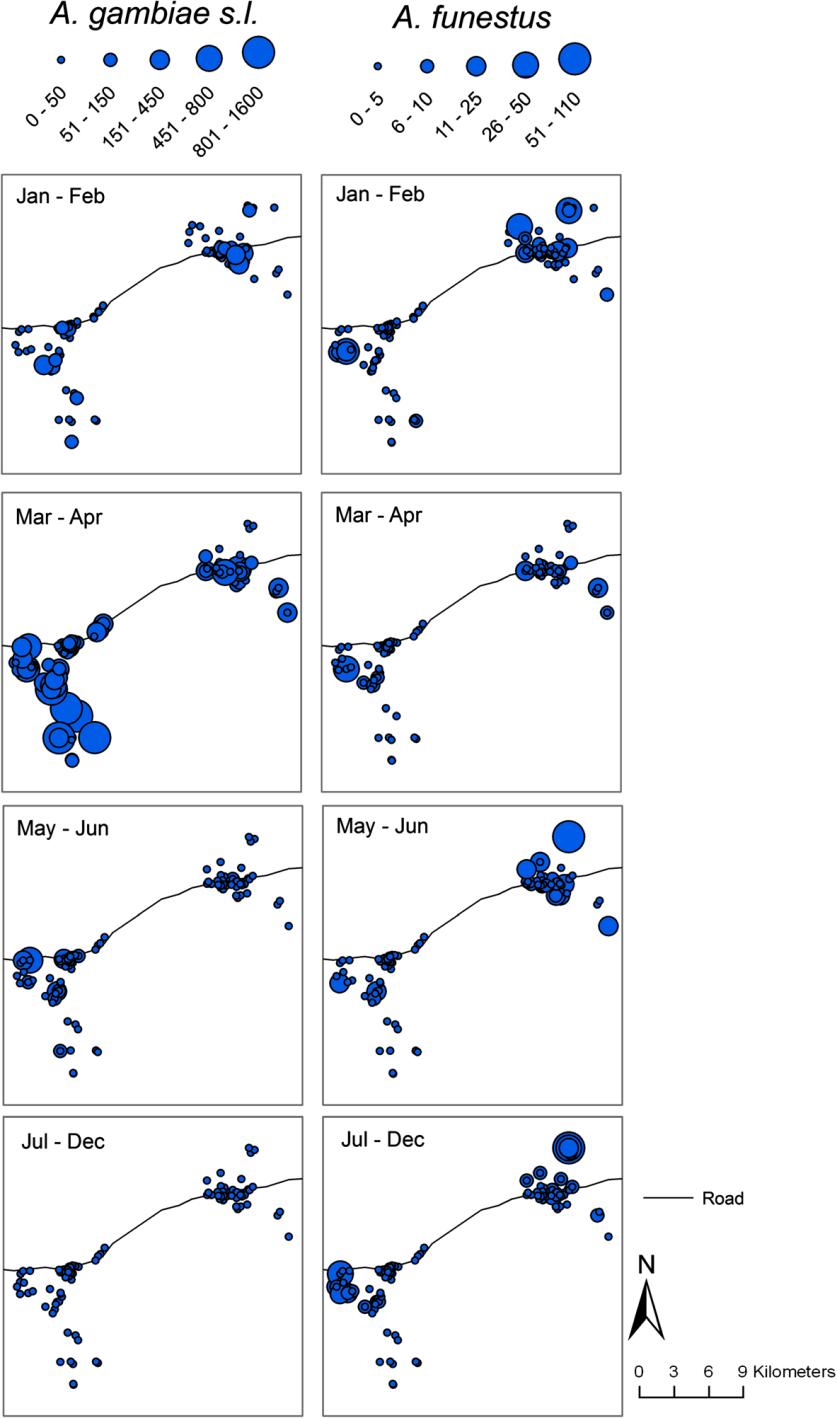
**Spatial distribution of *Anopheles gambiae *s.l. and *Anopheles funestus *densities over time in Namawala and Idete villages.** Note: Scale represents the total number of mosquitoes caught in all light trap sampling efforts for each household.

**Figure 4:**
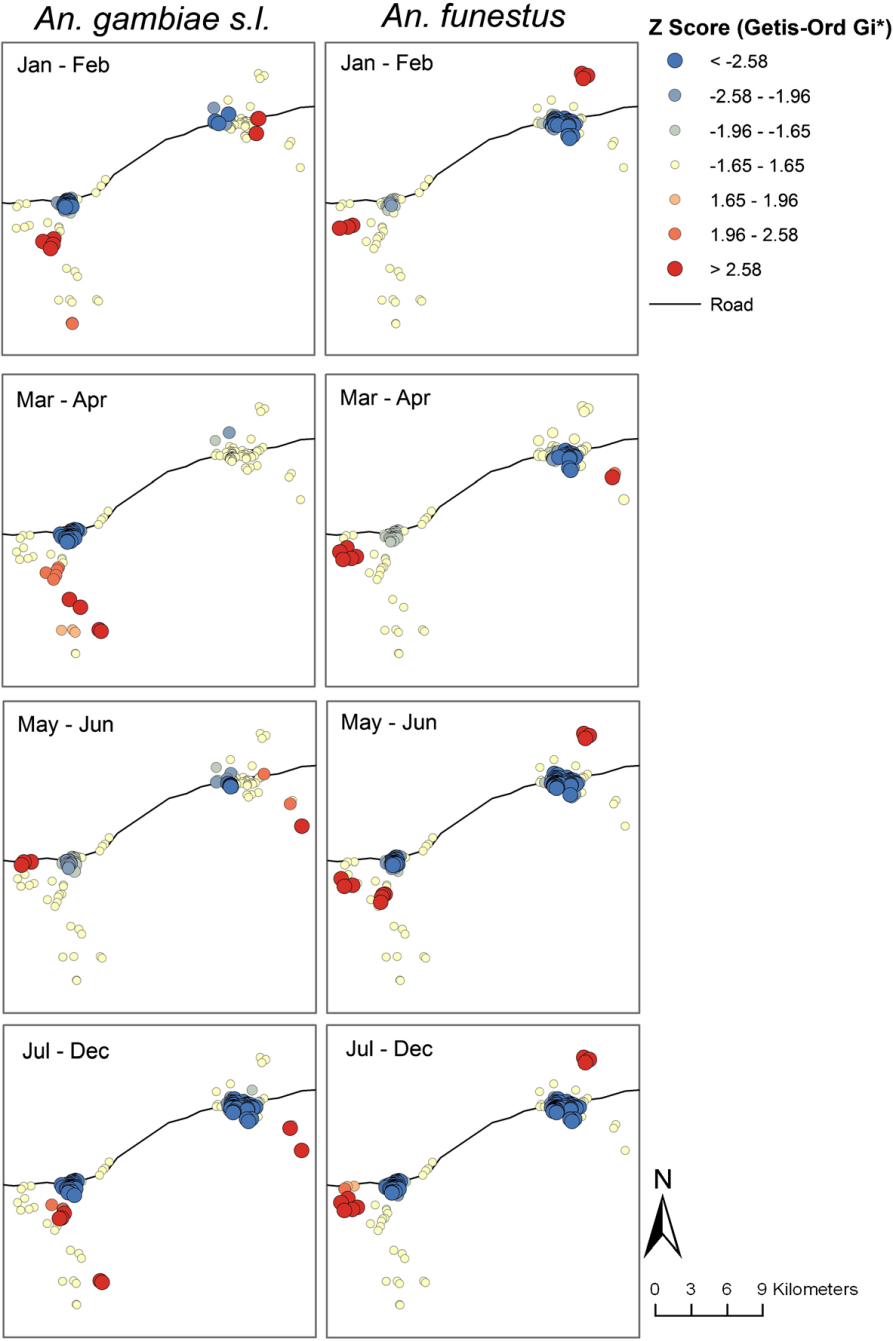
**Spatial clustering of *Anopheles gambiae *s.l. and *Anopheles funestus *densities over time in Namawala and Idete villages.** Note: The blue end of the scale represents clustering of households with low mosquito densities, where the red end of the scale represents high mosquito densities.

Extremely high densities of *An. gambiae* s.l. occurred during the wet season, especially during March to April; whereas the density of *An. funestus* was relatively consistent throughout the year (Figure 3 and [24]). Mapping the spatial patterns of *An. gambiae* s.l. and *An. funestus* densities indicated that households with high or low densities of anophelines tended to be closer together (Figure 3) and this was confirmed with the Moran’s *I* spatial analysis. For both *An. gambiae* s.l. and *An. funestus*, the existence of spatial autocorrelation, or clustering, of host-seeking densities was evidenced with positive *z* scores (Table 1). Specifically for *An. gambiae* s.l., clustering was evident in Namawala during all time periods, except May to June, and was significant overall. For *An. gambiae* s.l. in Idete, clustering was only significant during July to December. With regard to *An. funestus*, clustering was significant in both villages during all time periods, except March to April, and was significant overall. The age structure (parity) of *An. gambiae* s.l. and houses with sporozoite positive *An. gambiae* s.l. and *An. funestus* specimens were randomly distributed across the landscape (Table 2). Thus, the biting rate was the only component of the EIR which demonstrated any spatial autocorrelation, this is supported by Magbity and Lines [28]. The localities of high and low clusters of anopheline densities were identified with the *Z* scores computed by Getis-Ord Gi* (Figure 4). For *An. gambiae* s.l., there were nine households with high densities and 46 with low densities. For *An. funestus*, there were seven households with high densities and 96 with low densities.

**Table 1:** **Moran’s *I *spatial autocorrelation indices for *Anopheles gambiae *s.l. and *Anopheles funestus *densities over time in Namawala and Idete villages**

**Village and period**	***An. gambiae s.l.***			***An. funestus***		
	**Moran’s *I***	***z *score**	***p *value**	**Moran’s *I***	***z *score**	***p *value**
*Namawala*						
Jan-Feb	0.1020	4.8227	<0.0001	0.0907	4.3113	<0.0001
Mar-Apr	0.1938	10.0408	<0.0001	0.0357	2.2961	0.0216
May-Jun	0.0504	3.0473	0.0023	0.0973	5.3326	<0.0001
Jul-Dec	0.1776	9.1759	<0.0001	0.1820	9.3558	<0.0001
OVERALL	0.2007	10.3218	<0.0001	0.1583	8.1020	<0.0001
*Idete*						
Jan-Feb	−0.0089	0.6351	0.5254	0.0147	3.4600	0.0005
Mar-Apr	−0.0268	−1.5062	0.1320	−0.0033	1.2696	0.2042
May-Jun	−0.0026	1.3920	0.1639	0.0249	4.0918	<0.0001
Jul-Dec	0.0279	4.6912	<0.0001	0.0428	6.1554	<0.0001
OVERALL	−0.0228	−1.1972	0.2312	0.0440	6.4620	<0.0001

**Table 2:** **Moran’s *I *spatial autocorrelation indices for the sporozite positivity and parity of *Anopheles gambiae *s.l. and *Anopheles funestus *specimens collected in Namawala and Idete villages**

	**Moran’s *I***	***z *score**	***p *value**
**Sporozoite positive – *An. gambiae *s.l.**			
Namawala	0.0176	1.5925	0.1113
Idete	−0.0049	−0.9838	0.3252
**Sporozoite positive – *An. funestus***			
Namawala^a^	NA	NA	NA
Idete	−0.0128	0.3168	0.7514
**Parity – *An. gambiae *s.l.**			
Namawala	−0.0162	0.1216	0.9032
Idete	0.0058	1.3514	0.1766

^a^ No sporozoite positive *An. funestus* were detected in Namawala village.

**Figure 5:**
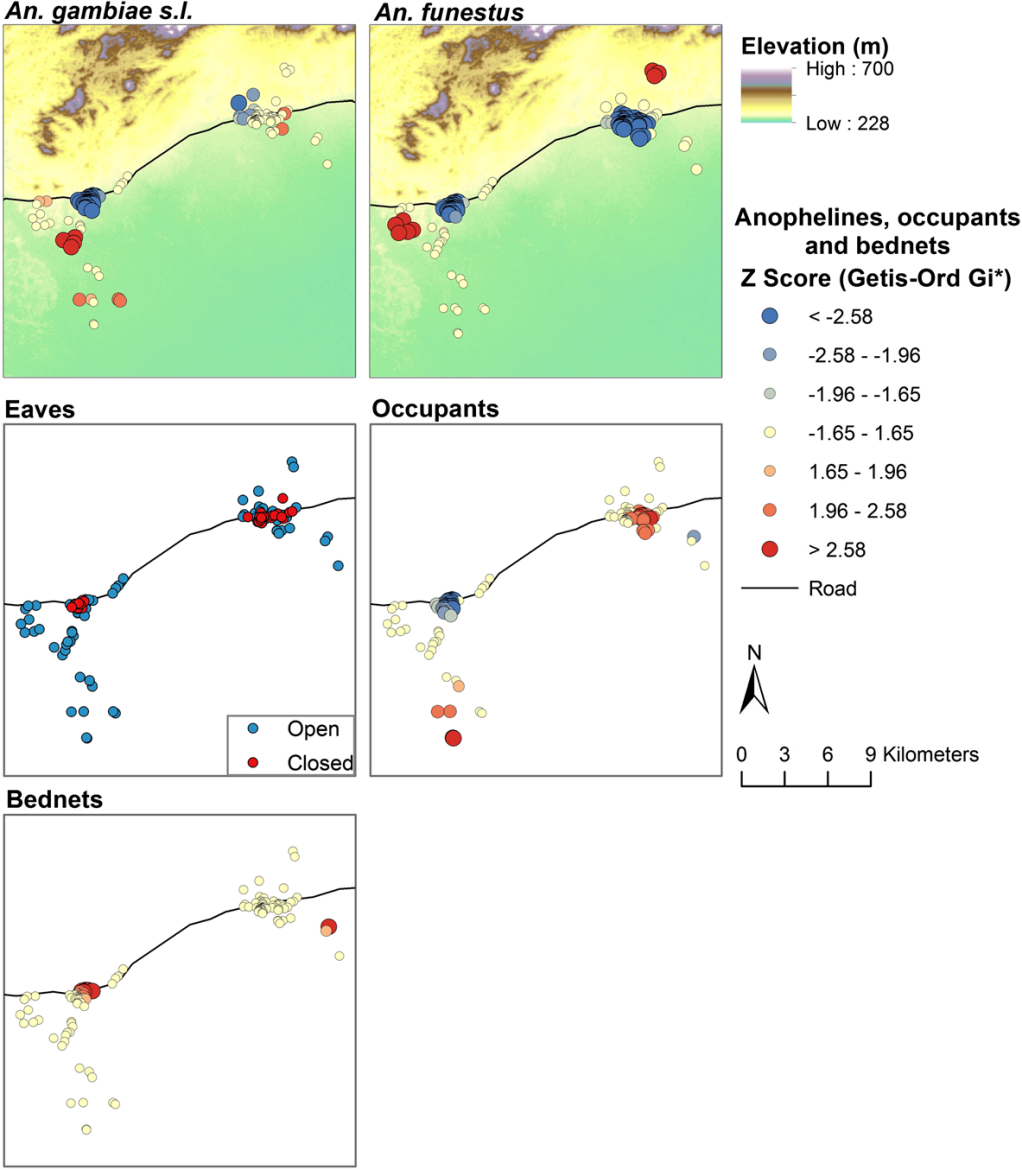
**The spatial clustering of *Anopheles gambiae *s.l. and *Anopheles funestus *densities computed over each month in 2008.** As well as the elevation profile, distribution of houses with closed/open eaves, and the spatial clustering of the number of household occupants and the number of LLINs per person. Note: The blue end of the scale represents clustering of households with low mosquito densities, where the red end of the scale represents high mosquito densities.

The influence of household characteristics on the localities of anopheline clustering was sequentially investigated (Table 3). The independent household characteristics that were correlated with distance from the village centre were: number of occupants (*t* = 2.662; *p* = 0.0087), elevation (*t* = −4.535; *p* = <0.0001) and eaves (*z* = 2.883; *p* = 0.0039) (see Figure 5). The mean distance of houses with open eaves to the centre of the village was 1,881 m, while the mean distance of houses with closed eaves was 629 m. The total number of bed nets owned by each household was not correlated with distance from the village centre (*t* = 0.440; *p* = 0.6604).

**Table 3:** **The mean characteristics of households that were in clusters of high or low densities of *Anopheles gambiae *s.l. and *Anopheles funestus***

		**Cluster mean**^**a**^	
**Species**	**Factor**	**Low**	**High**
***An. gambiae *s.l.**	Elevation (m)	286	264
	Eaves – Closed	23.94%	11.12%
	Occupants	3.56	5.77
	Bed nets/person	0.61	0.44
	Distance^b^ (m)	536	4355
***An. funestus***	Elevation (m)	284	286
	Eaves – Closed	31.25%	0.00%
	Occupants	4.57	3.28
	Bed nets/person	0.59	0.63
	Distance^b^ (m)	564	3770

^a^Clusters of households with high densities of anophelines were identified with *Z* scores >1.96, or *vice versa*, clustered households with low densities of anophelines were identified with *Z* scores < −1.96.

^b^The distance of each household from the village centre was calculated using ArcGIS software (ESRI, Redlands, CA, USA).

**Figure 6:**
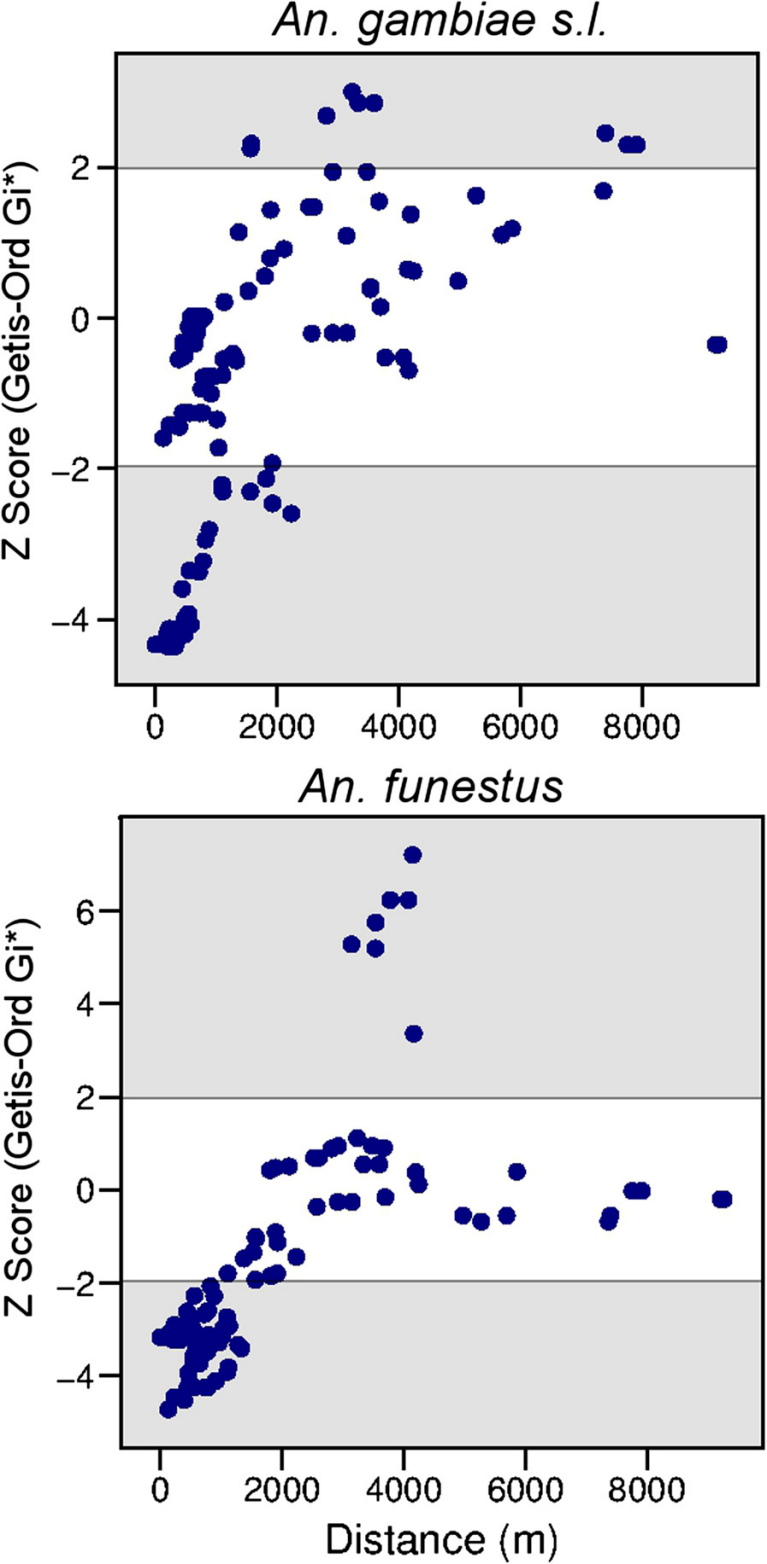
**Scatter plots comparing the spatial clustering of *Anopheles gambiae *s.l. and *Anopheles funestus *densities with distance from the village centre.** Spatial clustering is represented by the species specific Getis-Ord Gi* Z Score calculated for each household; clusters of households with high densities were identified with *Z* scores >1.96 (shaded area at top), or *vice versa*, clustered households with low densities of anophelines were identified with *Z* scores < −1.96 (shaded area at bottom).

The multivariate GLM examining the association between household characteristics and clustering of mosquitoes found that distance from the village centre significantly influenced the occurrence of anopheline clusters (Table 4, Figure 6). For both *An. gambiae* s.l. and *An. funestus*, houses with low densities were clustered in the semi-urban centre of Idete and Namawala, and there was almost no variability in the location of these low-density clusters over time. Conversely, houses with high densities of *An. gambiae* s.l. and *An. funestus* were clustered in the rural outskirts of both villages. There was some seasonal variability in the locality of the high-density clusters. Broadly, households located towards the periphery of each village had a higher chance of being located with a cluster of households that had higher densities of anophelines.

**Table 4:** **Multivariate statistical comparison of household factors with clustering of *Anopheles gambiae *s.l. and *Anopheles funestus *densities**

**Species**	**Factor**	***β***	**SE**	***p***
***An. gambiae *s.l.**	Elevation (m)	−0.032	0.013	0.0152*
	Eaves – Closed	−0.806	0.013	0.1421
	Occupants	0.376	0.084	<0.0001*
	Bed nets/person	0.411	0.552	0.4581
	Distance from centre (m)	0.003	0.001	0.0413*
	Distance from centre (m) × Elevation (m)	<0.001	<0.001	0.0065*
	Distance from centre (m) × Eaves – Closed	0.001	<0.001	0.0149*
	Distance from centre (m) × Occupants	<−0.001	<0.001	0.0008*
***An. funestus***	Elevation (m)	−0.058	0.015	<0.0001*
	Eaves – Closed	0.026	0.475	0.9622
	Occupants	−0.089	0.074	0.2293
	Bed nets/person	−0.241	0.481	0.6169
	Distance from centre (m)	−0.011	0.001	<0.0001*
	Distance from centre (m) × Elevation (m)	<0.001	<0.001	<0.0001*
	Distance from centre (m) × Eaves – Closed	<−0.001	<0.001	0.8498
	Distance from centre (m) × Occupants	<−0.001	<0.001	0.6153

The association of anopheline clusters (Getis-Ord Gi* *Z* Score) with household factors was compared with a generalized linear model (GLM) with a normal distribution.

With regard to the remaining household characteristics, the occurrence of *An. gambiae* s.l. clusters was also associated with elevation and the number of occupants; both of these factors interacted with distance from the village centre (Table 4). Elevation was negatively associated with clusters. Notably households with high densities of *An. gambiae* s.l. occurred in the south of Namawala, being on the flood-plain and in close association with larval habitats (Figure 5). The number of occupants in a household was positively associated with high *An. gambiae* s.l. densities. It is important to note that households with higher numbers of occupants were generally clustered outside of the semi-urban centres (Figure 5). For *An. funestus*, elevation also significantly influenced the location of clusters and the influence of this factor interacted with distance from the village centre. The influence of this interaction can be seen in Figure 6: the high-density houses have diverged from the general pattern for distance from the village centre.

Interestingly, the number of bed nets/person was not associated with the spatial clustering of either *An. gambiae* s.l. or *An. funestus*. This occurred because the bed nets were fairly evenly distributed across the landscape with minimal or no evidence of clustering (Figure 5). This represents the equity of the national bed net distribution system as it operated in the Kilombero Valley [36] and nationally [37]. The nets represented various distribution schemes and 46.8% of nets in use were either LLINs or had been treated within 1 year (for more detail see [24]).

## Discussion

Previously, models have demonstrated that the proportion of mosquitoes that are infectious is influenced by the age structure of the population. Specifically, the highest proportion of positive mosquitoes was modelled to be in localities with older mosquitoes, usually being the middle of villages and away from breeding sites [9]. This study did not find any statistical evidence for spatial clustering of the age structure of mosquitoes or the localities where sporozoite-infected, host-seeking female mosquitoes were caught. Consistent with other studies [38], of the two factors which comprise EIR (biting rate and sporozoite rate), only the biting rate expressed strong spatial heterogeneity. This study, along with the published literature [9-12] indicates that host-seeking mosquito densities are clustered and thus the risk of malaria infection at a household level is relatively more similar for close-by neighbours and not necessarily similar to the risk experienced by households further away but still situated in the same village. Such heterogeneous clustering of anopheline densities within villages [23,39,40] has usually been associated with the proximity to larval sites at this fine-scale level [12,18-21].

The number of household occupants positively influenced the densities of *An. gambiae* s.l. This is supported by previous research demonstrating that the number of household occupants does influence mosquito densities at an individual household level [15]. Thus the combined body odour of the many occupants may have attracted more mosquitoes to the area [41,42] and caused the host-seeking mosquitoes to aggregate [9]. In the current study, houses with more occupants, and also open eaves, tended to occur towards the periphery of the villages where higher densities of anophelines were also observed. Both the number of occupants and house construction are proxy indicators of socioeconomic status [43,44] and both have previously been associated with increased malaria risk [15,18,21,40,45,46]. Thus, this study demonstrates that as households are located towards the periphery of each village there is a gradient of increasing hazard (mosquito densities) and vulnerability (open eaves and larger households) which coincide in parallel from the village centre moving outwards.

For *An. gambiae* s.l., there was some mild variation in the locality of the high density clusters over time. This is most likely related to the ephemeral nature of larval habitats that changed drastically throughout the year. In the Kilombero Valley, the occurrence of *An. gambiae* s.l. larval sites is closely related to elevation: the flood plain occurs at lower elevations where the gradient of the land is gentler and water is able to pool and form larval sites, being one of the most influential factors associated with the location of high-density clusters [47]. For *An. funestus*, the localities of high-density clusters were localized with little spatial-temporal variability, as such it is plausible that the locality of the high clusters was related to the availability of larval sites. Especially since the larvae of *An. funestus* utilize large permanent or semi-permanent vegetated aquatic habitats such as stream pools and swamps [48]. For both *An. gambiae* s.l. and *An. funestus*, the locality of low-density clusters was consistent over time and may have been influenced by the closing of household eaves in the low-density clusters. The elevation of the study houses varied by less than 100 m (lowest house: 244 m; highest house: 334 m), thus it is unlikely that the temperature or relative humidity experienced by individual houses varied greatly; even though these factors are known to influence the distribution of vectors at broader scales [47,49].

Apart from the LLINs that were provided to the study houses to comply with ethical protocols, LLINs and other bed nets were fairly evenly distributed across the landscape and therefore did not contribute to within village spatial clustering of anopheline densities. Nonetheless, the distribution of LLINs has impacted on the community-wide transmission in the villages by reducing the host-seeking density, sporozoite rate and the EIR of the anopheline populations overall [24]. Particularly in this study area, the scale-up in LLINs has seen a dramatic reduction in the density of vectors which feed indoors during the middle of the night, such as *An. gambiae* s.s. [24,25]. Consequently, any residual transmission is maintained by mosquitoes which express exophagic feeding behaviours, such as *An. arabiensis*, which can feed outdoors at dusk or dawn [25,50,51]. Thus exists a need for complementary vector control interventions to help further suppress malaria transmission by targeting stages of the mosquito cycle, other than indoor biting, such as the larval stages, mating or sugar feeding [22]. The results of the current study highlight that such complementary vector control tools could be most effectively targeted to the periphery of villages where the households potentially have a higher hazard (mosquito densities) and vulnerability (open eaves and larger households) to malaria infection.

## Competing interests

The authors declare that they have no competing interests.

## Authors’ contributions

TLR designed the study, supervised the mosquito sampling protocol, performed the data analysis and wrote the first draft of the manuscript. DWL implemented the longitudinal mosquito sampling protocol and assisted with data entry and analysis. BK, WT and GFK contributed to the design of the field surveys and interpretation of the results. LKH supported the study design, data analysis and interpretation and contributed to the drafting of the manuscript. All authors have read and approved the final manuscript.

## References

[B1] WHO Global malaria control and elimination: report of a technical review 2008 Geneva: World Health Organization

[B2] WHO World malaria report 2011 2011 Geneva: World Health Organization

[B3] Bhattarai A Ali AS Kachur SP Mårtensson A Abbas AK Khatib R Al-mafazy A-w Ramsan M Rotllant G Gerstenmaier JF Molteni F Abdulla S Montgomery SM Kaneko A Björkman A Impact of artemisinin-based combination therapy and insecticide-treated nets on malaria burden in Zanzibar PLoS Med 2007 4 e309 10.1371/journal.pmed.0040309 17988171 PMC2062481

[B4] Ceesay SJ Casals-Pascual C Nwakanma DC Walther M Gomez-Escobar N Fulford AJC Takem EN Nogaro S Bojang KA Corrah T Jaye MC Taal MA Sonko AAJ Conway DJ Continued decline of malaria in The Gambia with implications for elimination PLoS One 2010 5 e12242 10.1371/journal.pone.0012242 20805878 PMC2923605

[B5] D’Acremont V Lengeler C Genton B Reduction in the proportion of fevers associated with Plasmodium falciparum parasitaemia in Africa: a systematic review Malar J 2010 9 240 10.1186/1475-2875-9-240 20727214 PMC2936918

[B6] O’Meara WP Mangeni JN Steketee R Greenwood B Changes in the burden of malaria in sub-Saharan Africa Lancet Infect Dis 2010 10 545 555 10.1016/S1473-3099(10)70096-7 20637696

[B7] Feachem R Sabot O A new global malaria eradication strategy Lancet 2008 371 1633 1635 10.1016/S0140-6736(08)60424-9 18374409

[B8] The malERA Consultative Group on Vector Control A research agenda for malaria eradication: vector control PLoS Med 2011 8 e1000401 21311587 10.1371/journal.pmed.1000401 PMC3026704

[B9] Smith DL Dushoff J McKenzie FE The risk of a mosquito-borne infection in a heterogeneous environment PLoS Biol 2004 2 1957 1964 10.1371/journal.pbio.0020368 PMC524252 15510228

[B10] Smith T Charlwood JD Takken W Tanner M Spiegelhalter DJ Mapping the densities of malaria vectors within a single village Acta Trop 1995 59 1 18 10.1016/0001-706X(94)00082-C 7785522

[B11] Trape JF Lefebvre-Zante E Legros F Ndiaye G Bouganali H Druilhe P Salem G Vector density gradients and the epidemiology of urban malaria in Dakar, Senegal Am J Trop Med Hyg 1992 47 181 189 1354414 10.4269/ajtmh.1992.47.181

[B12] Lindsay SW Armstrong Schellenberg JRM Zeiler HA Daly RJ Salum FM Wilkins HA Exposure of Gambian children to Anopheles gambiae malaria vectors in an irrigated rice production area Med Vet Entomol 1995 9 50 58 10.1111/j.1365-2915.1995.tb00116.x 7696688

[B13] Shaukat A Breman J McKenzie FE Using the entomological inoculation rate to assess the impact of vector control on malaria parasite transmission and elimination Malar J 2010 9 122 10.1186/1475-2875-9-122 20459850 PMC2890672

[B14] Kelly-Hope L McKenzie FE The multiplicity of malaria transmission: a review of entomological inoculation rate measurements and methods across sub-Saharan Africa Malar J 2009 8 19 10.1186/1475-2875-8-19 19166589 PMC2656515

[B15] Kirby MJ Green C Milligan PM Sismanidis C Jasseh M Conway DJ Lindsay SW Risk factors for house-entry by malaria vectors in a rural town and satellite villages in The Gambia Malar J 2008 7 2 10.1186/1475-2875-7-2 18179686 PMC2267476

[B16] Ogoma SB Lweitoijera DW Ngonyani H Furer B Russell TL Mukabana WR Killeen GF Moore SJ Screening mosquito house entry points as a potential method for integrated control of endophagic filariasis, arbovirus and malaria vectors PLoS Negl Trop Dis 2010 4 e773 10.1371/journal.pntd.0000773 20689815 PMC2914752

[B17] Kirby MJ Ameh D Bottomley C Green C Jawara M Milligan PM Snell PC Conway DJ Lindsay SW Effect of two different house screening interventions on exposure to malaria vectors and on anaemia in children in The Gambia: a randomised control trial Lancet 2009 6736 61078 10.1016/S0140-6736(09)60871-0 PMC3776946 19732949

[B18] Gunawardena DM Wickremasinghe AR Muthuwatta L Weerasingha S Rajakaruna J Senanayaka T Kotta PK Attanayake N Carter R Mendis KN Malaria risk factors in an endemic region of Sri Lanka, and the impact and cost implications of risk factor-based interventions Am J Trop Med Hyg 1998 58 533 542 9598437 10.4269/ajtmh.1998.58.533

[B19] Clarke SE Bøgh C Brown RC Walraven GEL Thomas CJ Lindsay SW Risk of malaria attacks in Gambian children is greater away from malaria vector breeding sites Trans R Soc Trop Med Hyg 2002 96 499 506 10.1016/S0035-9203(02)90419-0 12474476

[B20] Cohen J Ernst K Lindblade K Vulule J John C Wilson M Local topographic wetness indices predict household malaria risk better than land-use and land-cover in the western Kenya highlands Malar J 2010 9 328 10.1186/1475-2875-9-328 21080943 PMC2993734

[B21] Zhou G Munga S Minakawa N Githeko AK Yan G Spatial relationship between adult malaria vector abundance and environmental factors in western Kenya highlands Am J Trop Med Hyg 2007 77 29 35 17620627

[B22] Griffin JT Hollingsworth TD Okell LC Churcher TS White M Hinsley W Bousema T Drakeley CJ Ferguson NM Basáñez M-G Ghani AC Reducing Plasmodium falciparum malaria transmission in Africa: a model-based evaluation of intervention strategies PLoS Med 2010 7 e1000324 10.1371/journal.pmed.1000324 20711482 PMC2919425

[B23] Bousema T Drakeley C Gesase S Hashim R Magesa S Mosha F Otieno S Carneiro I Cox J Msuya E Kleinschmidt I Maxwell C Greenwood B Riley E Sauerwein R Chandramohan D Gosling R Identification of hot spots of malaria transmission for targeted malaria control J Infect Dis 2010 201 1764 1774 10.1086/652456 20415536

[B24] Russell TL Lwetiojera DW Maliti D Chipwaza B Kihonda J Charlwood JD Smith TA Lengeler C Mwanyangala MA Nathan R Knols BGJ Takken W Killeen GF Impact of promoting longer-lasting insecticide treatment of bednets upon malaria transmission in a rural Tanzanian setting with pre-existing high coverage of untreated nets Malar J 2010 9 187 10.1186/1475-2875-9-187 20579399 PMC2902500

[B25] Russell TL Govella NJ Azizi S Drakeley CJ Kachur SP Killeen GF Increased proportions of outdoor feeding among residual malaria vector populations following increased use of insecticide-treated nets in rural Tanzania Malar J 2011 10 80 10.1186/1475-2875-10-80 21477321 PMC3084176

[B26] Lines JD Curtis CF Wilkes TJ Njunwa KJ Monitoring human-biting mosquitoes (Diptera: Culicidae) in Tanzania with light-traps hung beside mosquito nets Bull Entomol Res 1991 81 77 84 10.1017/S0007485300053268

[B27] Mboera LEG Kihonda J Braks MA Knols BGJ Short report: Influence of centers for disease control light trap position, relative to a human-baited bed net, on catches of Anopheles gambiae and Culex quinquefasciatus in Tanzania Am J Trop Med Hyg 1998 59 595 596 9790436 10.4269/ajtmh.1998.59.595

[B28] Magbity EB Lines JD Marbiah MT David K Peterson E How reliable are light traps in estimating biting rates of adult Anopheles gambiae s.l. (Diptera: Culicidae) in the presence of treated bed nets? Bull Entomol Res 2002 92 71 76 12020364 10.1079/BER2001131

[B29] Killeen GF Tami A Kihonda J Okumu FO Kotas ME Grundmann H Kasigudi N Ngonyani H Mayagaya V Nathan R Abdulla S Charlwood JD Smith TA Lengeler C Cost-sharing strategies combining targeted public subsidies with private-sector delivery achieve high bednet coverage and reduced malaria transmission in Kilombero Valley, southern Tanzania BMC Infect Dis 2007 7 121 10.1186/1471-2334-7-121 17961211 PMC2211306

[B30] Gillies MT Coetzee M A supplement to the Anophelinae of Africa south of the Sahara (Afrotropical region) 1987 Johannesburg: South African Institute for Medical Research

[B31] Scott JA Brogdon WG Collins FH Identification of single specimens of the Anopheles gambiae complex by the polymerase chain reaction Am J Trop Med Hyg 1993 49 520 529 8214283 10.4269/ajtmh.1993.49.520

[B32] Burkot TR Williams JL Schneider I Identification of Plasmodium falciparum infected mosquitoes by a double antibody enzyme-linked immunosorbent assay Am J Trop Dis Hyg 1984 33 783 788 10.4269/ajtmh.1984.33.783 6385740

[B33] Gillies MT A modified technique for the age-grading of populations of Anopheles gambiae Ann Trop Med Parasitol 1958 52 261 273 13595554 10.1080/00034983.1958.11685867

[B34] Moran PAP The interpretation of statistical maps J R Stat Soc Series B Stat Methodol 1948 10 243 251

[B35] Getis A Ord JK The anlaysis of spatial association by use of distance statistics Geogr Anal 1992 24 189 206

[B36] Armstrong Schellenberg JRM Abdulla S Minja H Nathan R Mukasa O Marchant T Mponda H Kikumbih N Lyimo E Manchester T Tanner M Lengeler C KINET: a social marketing programme of treated nets and net treatment for malaria control in Tanzania, with evaluation of child health and long-term survival Trans R Soc Trop Med Hyg 1999 93 225 231 10.1016/S0035-9203(99)90001-9 10492745

[B37] Bonner K Mwita A McElroy P Omari S Mzava A Lengeler C Kaspar N Nathan R Ngegba J Mtung’e R Brown N Design, implementation and evaluation of a national campaign to distribute nine million free LLINs to children under five years of age in Tanzania Malar J 2011 10 73 10.1186/1475-2875-10-73 21453519 PMC3078903

[B38] Magbity EB Lines JD Spatial and temporal distribution of Anopheles gambiae s.l. (Diptera: Culicidae) in two Tanzanian villages: implications for designing mosquito sampling routines Bull Entomol Res 2002 92 483 488 17598299 10.1079/ber2002200

[B39] Brooker S Clarke S Njagi JK Polack S Mugo B Estambale B Muchiri E Magnussen P Cox J Spatial clustering of malaria and associated risk factors during an epidemic in a highland area of western Kenya Trop Med Int Health 2004 9 757 766 10.1111/j.1365-3156.2004.01272.x 15228485

[B40] Gamage-Mendis AC Carter R Mendis C De Zoysa AP Herath PR Mendis KN Clustering of malaria infections within an endemic population: risk of malaria associated with the type of housing construction Am J Trop Med Hyg 1991 45 77 85 1867350 10.4269/ajtmh.1991.45.77

[B41] Takken W Knols BGJ Odor-mediated behavior of Afrotropical malaria mosquiotes Annu Rev Entomol 1999 44 131 157 10.1146/annurev.ento.44.1.131 9990718

[B42] Kelly DW Why are some people bitten more than others? Trends Parasitol 2001 17 578 581 10.1016/S1471-4922(01)02116-X 11756041

[B43] Somi MF Butler JR Vahid F Njau JD Kachur SP Abdulla S Use of proxy measures in estimating socioeconomic inequalities in malaria prevalence Trop Med Int Health 2008 13 354 364 10.1111/j.1365-3156.2008.02009.x 18397398

[B44] Somi MF Butler JRG Vahid F Njau J Kachur SP Abdulla S Is There evidence for dual causation between malaria and socioeconomic status? Findings from rural Tanzania Am J Trop Med Hyg 2007 77 1020 1027 18165515

[B45] Ghebreyesus TA Haile M Witten KH Getachew A Yohannes M Lindsay SW Byass P Household risk factors for malaria among children in the Ethiopian highlands Trans R Soc Trop Med Hyg 2000 94 17 21 10.1016/S0035-9203(00)90424-3 10748890

[B46] Haddow AJ The mosquito fauna and climate of native huts at Kisumu, Kenya Bull Entomol Res 1942 33 91 142 10.1017/S0007485300026389

[B47] Kelly-Hope L Hemingway J McKenzie FE Environmental factors associated with the malaria vectors Anopheles gambiae and Anopheles funestus in Kenya Malar J 2009 8 268 10.1186/1475-2875-8-268 19941637 PMC2793260

[B48] Sinka M Bangs M Manguin S Coetzee M Mbogo C Hemingway J Patil A Temperley W Gething P Kabaria C Okara R Van Boeckel T Godfray HC Harbach R Hay S The dominant Anopheles vectors of human malaria in Africa, Europe and the Middle East: occurrence data, distribution maps and bionomic precis Parasit Vectors 2010 3 117 10.1186/1756-3305-3-117 21129198 PMC3016360

[B49] De Souza D Kelly-Hope L Lawson B Wilson M Boakye D Environmental factors associated with the distribution of Anopheles gambiae s.s in Ghana; an important vector of lymphatic filariasis and malaria PLoS One 2010 5 e9927 10.1371/journal.pone.0009927 20360950 PMC2847902

[B50] Bayoh MN Mathias D Odiere M Mutuku F Kamau L Gimnig J Vulule J Hawley W Hamel M Walker E Anopheles gambiae: historical population decline associated with regional distribution of insecticide-treated bed nets in western Nyanza Province, Kenya Malar J 2010 9 62 10.1186/1475-2875-9-62 20187956 PMC2838909

[B51] Mutuku F King C Mungai P Mbogo C Mwangangi J Muchiri E Walker E Kitron U Impact of insecticide-treated bed nets on malaria transmission indices on the south coast of Kenya Malar J 2011 10 356 10.1186/1475-2875-10-356 22165904 PMC3322380

